# Generalized Peritonitis Requiring Re-operation After Leakage of Omental Patch Repair of Perforated Peptic Ulcer

**DOI:** 10.4103/1319-3767.77243

**Published:** 2011

**Authors:** Hemmat Maghsoudi, Alireza Ghaffari

**Affiliations:** Department of Surgery, Faculty of Medicine, University of Medical Sciences of Tabriz, Iran

**Keywords:** Generalized peritonitis, omental patch, perforated peptic ulcer

## Abstract

**Background/Aim:**

Peptic ulcer perforations are a common emergency, but available literature is silent on the exact definition, incidence, management, and complications of peritonitis due to omental patch leakage.

**Patients and Methods:**

Retrospective data were collected on 422 patients who underwent omental patch repair of perforated peptic ulcer between March 20, 1999 and March 20, 2006. The definitive diagnosis of perforated peptic ulcer and omental patch leakage was obtained at surgery.

**Results:**

Seventeen (4%) patients experienced generalized peritonitis due to omental patch leakage. Mean age was 60.6 years. Mortality rate was 29.4%, and the mean hospital stay was 23.6 days. Delay in surgical approach, shock on admission, and age were all significantly associated with increased mortality.

**Conclusions:**

Peritonitis due to omental patch leakage can result in significant morbidity and mortality. The most common causes of omental patch leakage and operative procedures were unknown and reinsertion of omentum, respectively. Factors such as shock on admission or delayed surgery, have significantly contributed to fatal outcomes and need careful attention.

The first clinical description of perforated peptic ulcer was made by Crisp in 1843. The features of the disease and of patients affected by it, have changed ever since. During the nineteenth century, ulcer perforation was a rare disease that occurred mainly in young women with the perforation located near the cardia of the stomach.[1,2] During the early twentieth century, ulcer perforation increased in incidence, being commonly situated in the duodenum in middle-aged men (peaking for men around the 1950s, though it appears to continue to increase for women).[2,3] The management of peptic ulcer disease has evolved over the decades, due to advances in operative techniques, bacteriology and pharmacology. There has been a marked decrease in elective surgery for peptic ulcer disease (PUD) following introduction of medical therapies including H2-receptor antagonist, and more recently proton pump inhibitors with or without antibiotic for *H. pylori* eradication. By contrast, the number of acute complications e.g. ulcer perforation and bleeding required emergency surgery, have remained quantitatively constant.[4–6] Peptic ulcer perforation is a serious complication which affects almost 10% of PUD patients.[6] The revolution in ulcer treatment that occurred with the discovery of the role of *H. pylori* has not yet led to any detectable changes in the incidence of ulcer perforation.[2,7] The potential for prevention thus lies in better understanding of causal factors which have not been known till lately, but apparently differ somewhat from those of uncomplicated ulcers.[2,8,9] Generalized peritonitis after omental patch repair has not been reported in the literature. This report describes a retrospective study of the occurrence of generalized peritonitis requiring re-operation after omental patch repair of perforated peptic ulcer in 17 patients. We analyzed operative findings and procedures and evaluated hospital morbidity and mortality.

## PATIENTS AND METHODS

Between March 20, 1999 and March 20, 2006, 422 consecutive patients with perforated peptic ulcer (established intra-operative) underwent operation or re-operation at two large hospitals in Tabriz, Iran (Imam hospital and Sina hospital).

Using a standardized data collection form, the following information was obtained. During this time, 422 perforated peptic ulcers were operated by 10 different surgeons. All patients were treated exclusively by open surgical approach. No patients have been treated by laparoscopy (because of lack of experience and infrastructure).

**Figure 1:**
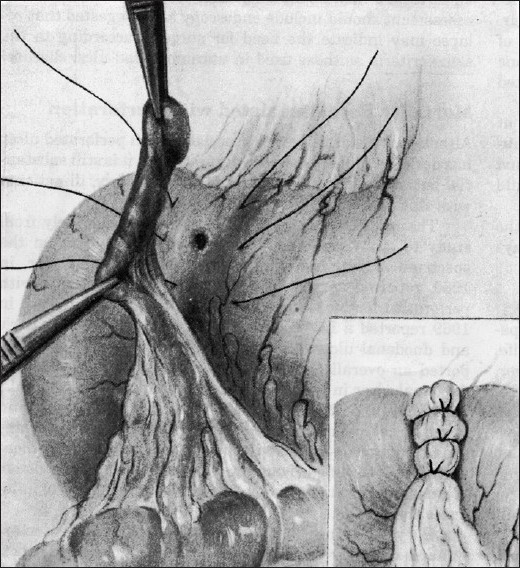
The method of omental patch fashioning

The diagnosis of peritonitis due to omental patch leakage was based on clinical features, routine laboratory tests, and radiological findings (i.e, plain abdominal X-ray and abdominal CT scan in all cases, if required). Invariably, the definitive diagnosis of perforated peptic ulcer and omental patch leakage was obtained at surgery. The method of omental patch repair is shown in Figure 1. The technique of omentopexy was essentially the same in all the cases – a total of three or four seromuscular interrupted sutures (silk) were placed onto the normal, healthy duodenum on either side of the perforation, a strand of well-vascularized omentum was placed directly onto the perforation, and the sutures were knotted above this. No attempt was made to close the perforation prior to placing the omentum as a graft. Simple patch closure is followed by *H. pylori* eradication for reduction of ulcer recurrence rate. Data were obtained on year of procedure, surgeon, length of stay, operative details and findings, size of perforation, generalized peritonitis and its management. Patients were excluded if the perforation was due to malignant disease or trauma. Local infections and local abscess were excluded from this study. No cases of anterior and posterior ulcers or multiple perforations were encountered. An open surgical approach was performed leading to a non-definitive operation (omental patch) in all patients. Patients were excluded if the operation was other than omental patch. All operations were performed by the same surgical staff whose colleagues were well trained in gastrointestinal surgery. Intravenous fluids, nasogastric decompression, intravenous antibiotics, analgesics, and careful monitoring and support of hemodynamics were instituted in the immediate postoperative period in all patients. The nasogastric tube was removed upon return of gastrointestinal transit, and feeding slowly begun. Proton pump inhibitors were used throughout the perioperative period, and treatment for *H. pylori* eradication was instituted immediately after the operative procedure and continued for two-four weeks when infection with this organism is suspected or documented. Results of treatment were confirmed later (six-eight weeks) with upper gastrointestinal endoscopy.[10]

The time between presumed perforation and surgery was considered delayed if longer than 12 h. The time between omental patch leak peritonitis and re-operation was divided to first 24 h and >24 h.

Data handling and analysis were performed with SPSS software for Windows, version 10.5 (SPSS Inc., Chicago, IL) for Chi-square test, Fisher’s exact test, or Student t-test for unequal variance where appropriate. *P* value of less than 0.05 was considered as statistically significant.

## RESULTS

**Figure 2:**
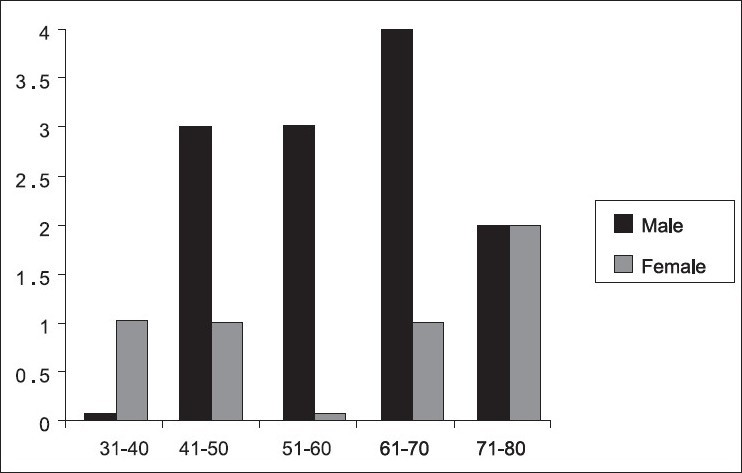
Distribution of patients according to age and sex

**Figure 3:**
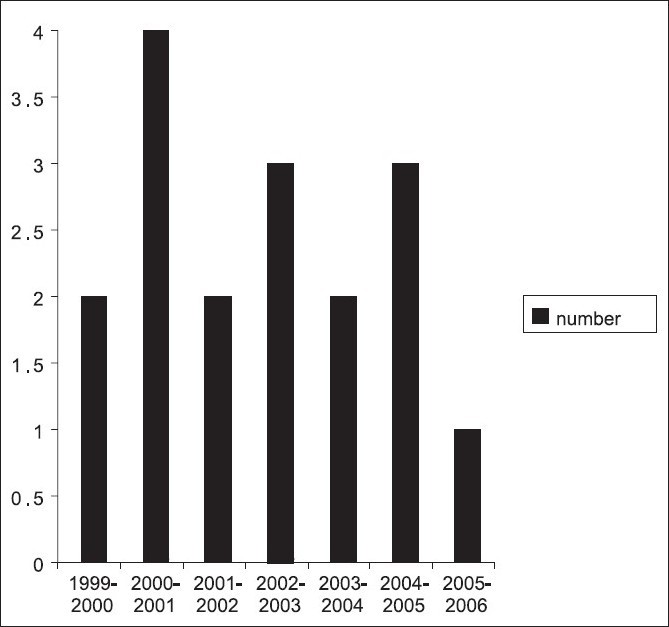
Seven-year distribution of 17 cases of peritonitis due to omental patch leakage

During the study period, 422 (375, 88.9% duodenal and prepyloric [gastric], 47, 11.1%) perforated peptic ulcer cases were operated by 10 different surgeons at the two hospitals. Seventeen (4%, all of them were perforated duodenal and prepyloric peptic ulcer) experienced generalized peritonitis after omental patch repair. In our study, no perforated gastric ulcer cases experienced generalized peritonitis after omental patch repair. The mean number of generalized peritonitis cases per surgeon was 1.7 (range 0 to 3). The incidence of omental patch leakage peritonitis remained constant throughout the study period. Five (29.4%) were females, and 12 (70.6%) were males. Sex and age distribution are shown in [Figure 2]. There was a variable trend in the number of patients during the seven-year study period [Figure 3]. Mean age was 60.6 years (range 39 to 80, SD: 12.87). Previous history of PUD was found in six (35.3%) patients and two (11.8%) patients were hypotensive on admission. The time between omental patch leakage and re-operation were within 24 h in 70.6% (12 of 17 patients) and > 24 h in 29.4% (5 of 17 patients). The analysis of factors associated with mortality in 17 patients undergoing surgery for peritonitis due to omental patch leakage compared with patients without leakage is depicted in Table 1. Of the 17 patients, 5 died yielding an overall mortality rate of 29.4%. The presence of one or more associated disease, delay in surgical approach (because difficulty of diagnosis in some patients), shock on admission, post-operative abdominal complications and age were all significantly (*P*<0.05) associated with increased mortality in patients undergoing surgery for PPU. By contrast, sex, previous history, site of peptic ulcer (duodenal or prepyloric), and the development of postoperative general complications were not associated with increased mortality.

**Table 1:** Analysis of factors associated with mortality in 17 patients undergoing surgery for peritonitis due to omental patch leakage compared with 405 patients without leakage

	17 patients with leakage			405 patients without leakage	
	Number	Mortality (%)	*P* value	Number of patients	Mortality
Male: female	12:5	33.3 *vs*. 20	NS	289:116	0
Age (<65: >65 years)	10:7	10 *vs*. 57	0.04	306:99	0
Previous ulcer history (yes:no)	11:6	27.2vs.33.3	NS	262:143	0
Delayed operations (yes:no)	Within 24h: 5, >24h: 12	40 vs. 25	0.04	<12h: 183, >12h:222	0
Size of perforation (<1 cm: 1-3 cm)	10:7	20% *vs*. 42.86%	NS	285:120	0
Shock on admission (yes: no)*	2:15	100 vs. 20	0.01	71:334	0

Data analyzed by χ^2^ test and

* Fisher’s exact test

It was noted that the omental patch had gangrenous appearance in five patients and partial or complete separation of omental patch in all patients who underwent relaparotomy. Causes of omental patch leakage were unknown in 12 patients. No patients were receiving steroids or chemotherapy or radiotherapy. Thirteen patients underwent reinsertion of omental patch and subhepatic drainage after laparotomy and abdominal cavity irrigation; one patient underwent jejunal serosal patch and drainage and abdominal cavity irrigation; and three patients underwent subhepatic drainage by Foley’s catheter after laparotomy and abdominal cavity irrigation [Table 2]. Catheters were removed at least three weeks after surgery, when tract of catheters was usually walled off. Patients of peritonitis due to omental patch leakage had a significantly prolonged hospital stay (mean 23.6 days SD; 7.38, range 14 to 40 days). Distribution and characteristics of 17 patients according to size of perforations are presented in Table 3. Operative procedures are shown in Table 2. Five patients (four male and one female) died from sepsis after surgery. No mortality was seen in the 405 patients who did not experience generalized peritonitis after omental patch repair.

**Table 2:** Surgical procedures in 17 patients with peritonitis occurring after omental patch leakage

Surgical procedure	Number of patients
Subhepatic drainage and abdominal cavity irrigation	3
Laparotomy and reinsertion of omental patch and subhepatic drainage	13
Jejenal serosal patch and drainage and abdominal cavity irrigation	1

**Table 3:** Distribution and characteristics of 17 patients according to size of perforation and time between omental patch leakage and re-operation

Patient data	Less than 1 cm	1 cm – 3 cm	First 24h	>24h
Number of cases	10(10 of 318)	7 (7 of 104)	12	5
Average age	64.2	55.57	55.3	65.7
Male/female	7: 3	5: 2	8:4	4:1
Average duration of symptoms	2.5 days	1.5	-	-
Mortality	2 (20%)	3 (42.86%)	1	4
Post-operative hospital stay	23.3 days	22.7 days	21.8	24.3

## DISCUSSION

Peptic ulcer perforation is a common surgical emergency in our part of the world.

The management of perforated peptic ulcer disease has been debated for several decades. Micklicz introduced closure of perforation by suture in 1880 when he closed a gastric ulcer perforation.[2,11] Non-operative management for perforated peptic ulcer disease (PUD) was described in 1935 by Wangensteen and is still applicable in specific conditions.[2,12] The first two cases of primary gastric resection for ulcer procedure were described in 1919 by Von Haberer.[13] In 1937, Roscoe Graham described simple closure of duodenal perforation.[14] Recognition of a high rate of both, recurrent symptoms and ulcer, with simple closure led many authors to recommend a definitive ulcer curative procedure.[2,15–21] Introduction of antibiotics in 1950’s reduced the rate of post-operative complications and deaths.[2,22] In our study, three operative procedures were used for the management of peritonitis due to omental patch leakage. However, management types are rarely recorded in the literature. In our study, 29.4% of the patients died. According to our knowledge, the mortality rate of peritonitis due to omental patch leakage has rarely been reported. There was no significant correlation between size of perforation and occurrence of peritonitis in our patients. The results of omental patch in small and large sized perforations in the present series give statistically similar results. The leakage rates and mortality of the two groups after omental patch repair remain comparable, thereby suggesting that this may be considered as the procedure of choice in all perforations upto a size 3 cm. This finding is similar to the findings of other studies.[23–25]

Our study revealed that technical error (tight suture of omentum) can cause gangrene of omentum and leakage (29.4% of our patients). This cause has not been reported in the literature. Other technical factors, such as omentum size and method of fashioning, did not contribute to leakage peritonitis in our series.

Several factors might contribute to increased post-operative leakage and mortality in patients with PPU.[6,26] Severely diseased and scarred perforation may preclude adequate closure. Leaking omental patch is most likely to occur when an attempt is made to carry out primary closure in a very large indurated perforation, especially in the stomach.[27] Re-operation is required in all cases of post-operative leakage peritonitis, but nature of the surgical intervention must be carefully judged. Rarely is it possible to successfully close a leaking omental patch.[27] But, in our study, 76.5% (13 of 17) patients were amenable for reclosure with omental patch. If possible, a suction catheter should be applied to the disrupted area in the duodenum and secured in place with non-absorbable suture material reinforced with omentum. Additional measures include the use of a disposable ileostomy bag over the drainage tract site for protection of the skin and to measure the fluid volume loss. Vigorous supportive therapy is stressed during the post-operative period and consists of wide-spectrum antibiotics along with total parenteral nutrition. Although the mortality and morbidity is high following an omental patch disruption, with prompt diagnosis and intervention, prognosis in most cases is favorable.[27]

Perforation has been found to be a major complication of PUD with a mortality rate ranging from 6% to 31%.[6,22–24,28] Age of patients with PPU has been gradually increasing over the last years.[22,23,28] In this series, an age > 65 years tended to be associated with increased mortality. This finding is in line with other studies in which older patients frequently had associated diseases, or were more likely to be receiving NSAID treatment.[6,24,28] In accordance with other studies,[6,26,29], we could not relate that men were associated with a greater mortality rate. Also, there was no significant difference in mortality rate in patients with or without previous ulcer history. Such apparent discrepancies might be explained by the characteristics of patients included in the study, and/or by different age or different surgical procedures.[6,30,31] This study confirmed the previous observations[29–34] that shock on admission and delayed operation were both associated with a greater mortality rate.

Perforated ulcers may be managed by local measures, although simple apposition of the ulcer has been described,[34] these may be technically difficult to perform because of surrounding induration of the tissue. The authors do not recommend it.

It is likely that leakage is an uncommon consequence of omental patch. Peritonitis due to omental patch leakage results in considerable mortality, morbidity, and excessive hospital stays. Although peritonitis after omental patch leakage is uncommon, many surgeons will experience this complication at least once during their professional careers. The present study did not aim to address the optimum management of omental patch leakage; however, it did highlight the significant morbidity and mortality that can arise with the use of omental patch.

The omental patch is simple, can be performed in a relatively short time, and remains dependable even for the closure of large sized perforations (i.e. perforations up to 3 cm in size)[35] If operative closure proves very difficult and there are serious concerns about possible continued leakage, the addition of a feeding jejunostomy and placement of a tube drain in the Morrison’s space may offer a further sense of ‘’security” to the operating surgeon, keeping the option of maintaining the nutrition of the patient as well as creating a controlled duodenal fistula in case of a post-operative leak.[35] Incomplete closure of the perforation can result in persistent leakage and continued peritonitis. It may be useful—though it is necessary in only a minority of cases before abdominal closure—to assess the adequacy of the repair intraoperatively by instilling saline with methylene blue through the nasogastric tube near the perforation, then compressing the duodenum beyond the perforation and looking for any fluid leakage from the repaired hole. To keep from devascularizing the omental pedicle, the sutures should not be tied too tightly. If the perforation is large (> 1 cm), the surgeon may consider incorporating the perforation into a pyloroplasty. In addition, genetic and personal factors should be studied.
